# Machine learning evaluation of LV outflow obstruction in hypertrophic cardiomyopathy using three-chamber cardiovascular magnetic resonance

**DOI:** 10.1007/s10554-022-02724-7

**Published:** 2022-10-06

**Authors:** Manisha Sahota, Sepas Ryan Saraskani, Hao Xu, Liandong Li, Abdul Wahab Majeed, Uxio Hermida, Stefan Neubauer, Milind Desai, William Weintraub, Patrice Desvigne-Nickens, Jeanette Schulz-Menger, Raymond Y. Kwong, Christopher M. Kramer, Alistair A. Young, Pablo Lamata, the HCMR investigators

**Affiliations:** 1 Department of Biomedical Engineering, King’s College London, 1 Lambeth Palace Rd, London, SE1 7EU UK; 2 Division of Cardiovascular Medicine, Radcliffe Department of Medicine, University of Oxford, Oxford, UK; 3 Cardiovascular Institute, Cleveland Clinic, Cleveland, OH USA; 4 MedStar Heart and Vascular Institute, Washington, DC USA; 5 National Heart, Lung, and Blood Institute, Bethesda, MD USA; 6 ECRC and Department of Cardiology, HELIOS Klinik Berlin-Buch, Clinic for Cardiology and Nephrology, DZHK Partnersite Berlin, Charité Medical University Berlin, Berlin, Germany; 7 Cardiovascular Division, Department of Medicine and Department of Radiology, Brigham and Women’s Hospital, Boston, MA USA; 8 Cardiovascular Division, University of Virginia Health, Charlottesville, VA USA

**Keywords:** Hypertrophic cardiomyopathy, Atlas shape analysis, LV outflow tract obstruction

## Abstract

Left ventricular outflow tract obstruction (LVOTO) is common in hypertrophic cardiomyopathy (HCM), but relationships between anatomical metrics and obstruction are poorly understood. We aimed to develop machine learning methods to evaluate LVOTO in HCM patients and quantify relationships between anatomical metrics and obstruction. This retrospective analysis of 1905 participants of the HCM Registry quantified 11 anatomical metrics derived from 14 landmarks automatically detected on the three-chamber long axis cine CMR images. Linear and logistic regression was used to quantify strengths of relationships with the presence of LVOTO (defined by resting Doppler pressure drop of > 30 mmHg), using the area under the receiver operating characteristic (AUC). Intraclass correlation coefficients between the network predictions and three independent observers showed similar agreement to that between observers. The distance from anterior mitral valve leaflet tip to basal septum (AML-BS) was most highly correlated with Doppler pressure drop (R^2^ = 0.19, p < 10^–5^). Multivariate stepwise regression found the best predictive model included AML-BS, AML length to aortic valve diameter ratio, AML length to LV width ratio, and midventricular septal thickness metrics (AUC 0.84). Excluding AML-BS, metrics grouped according to septal hypertrophy, LV geometry, and AML anatomy each had similar associations with LVOTO (AUC 0.71, 0.71, 0.68 respectively, p = ns), significantly less than their combination (AUC 0.77, p < 0.05 for each). Anatomical metrics derived from a standard three-chamber CMR cine acquisition can be used to highlight risk of LVOTO, and suggest further investigation if necessary. A combination of geometric factors is required to provide the best risk prediction.

**Supplementary Information:**

The online version contains supplementary material available at 10.1007/s10554-022-02724-7.

## Introduction

Hypertrophic cardiomyopathy (HCM) is a highly complex genetic disorder, characterised by the presence of left ventricular hypertrophy (LVH) without ventricular dilatation, that cannot be otherwise explained by abnormal loading. It is typically inherited as an autosomal dominant trait [1] with an estimated prevalence of 1 in 500 in the general population [2]. Dynamic left ventricular outflow tract obstruction (LVOTO) occurs in 20–25% of cases [3–5] leading to high wall stress and adverse outcomes [6].

Typically, Doppler transthoracic echocardiography is used for LVOTO evaluation, where a resting peak pressure drop (commonly referred to as “*gradient*”) ≥ 30 mmHg is indicative of LVOTO [4]. However, echocardiography can be limited by operator dependence, beam misalignment and poor acoustic windows, as well as assumptions in the Bernoulli estimation of pressure drops which are typically violated [7, 8]. Cardiovascular magnetic resonance (CMR) imaging enables accurate estimation of morphology and is routinely performed in HCM patients [9–14]. Recent guidelines recommend CMR for diagnosis, risk prediction, and preprocedural planning for septal reduction in HCM patients, with regular 3–5 year CMR imaging in cases where echocardiography is inadequate [35]. CMR and echocardiography are seen to be synergistic in HCM evaluation [35]. A three-chamber (3CH) long axis cine is typically acquired to evaluate LVOT morphology; however, the relationships between 3CH CMR and Doppler echocardiography have not been assessed.

CMR may also provide useful information on the mechanisms of LVOTO, which are thought to include three main contributions: (i) LV hypertrophy, including basal septal thickness, (ii) mitral valve interplay, including its systolic anterior motion (SAM) and and/or elongation of the anterior mitral leaflet (AML), and (iii) LV chamber morphology, including anterior displacement of the papillary muscles [15–18].

In this study we developed deep learning algorithms to provide fast and accurate detection of anatomical landmarks in 3CH long-axis CMR images to assess the extent to which landmark distances can be reliably estimated. We sought to evaluate the relationships between landmark dimensions and Doppler echocardiographic pressure drop in a large cohort of HCM patients drawn from the HCM Registry study [19, 20]. We hypothesised that individual landmark distances are significantly correlated with LVOTO, and that different mechanistic factors, using combinations of landmark distances, are independently related to LVOTO.

## Methods

### Overview

**Fig. 1:**
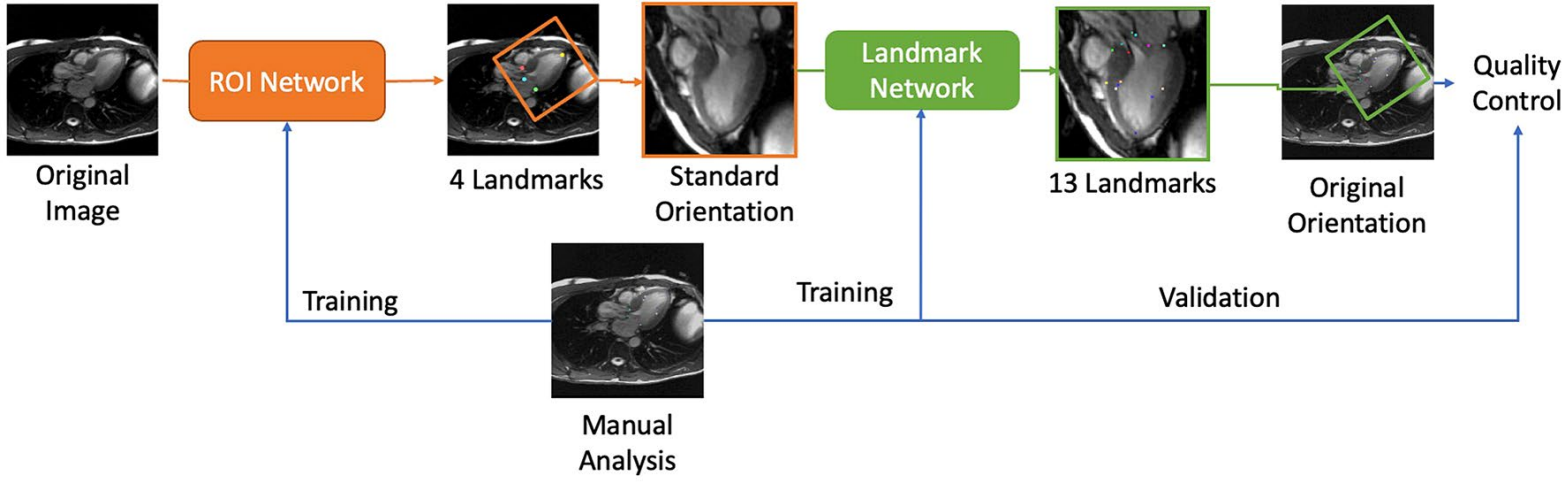
The image analysis pipeline

Figure 1 shows the analysis pipeline used in this study. Data from the HCM Registry study were used to train and validate a landmark detection network consisting of two steps. Firstly, an initial region of interest network was trained to detect the location and orientation of the heart in 3CH images. This was used to crop and rotate the images into a standard orientation. Then a landmark detection network was trained to evaluate the cropped rotated images. Finally, all cases were evaluated with the landmark detection model. The following sections describe each step in the process.

### Subjects and data acquisition

The HCM Registry Study is a prospective registry consisting of 2750 patients recruited from 44 European and North American sites. Exclusion and inclusion criteria have been detailed previously [19, 20]. Briefly, patients were excluded if known to have other infiltrative/hypertrophic cardiomyopathies, older than 65 years, prior septal myectomy or alcohol septal ablation, prior myocardial infarction or known coronary artery disease, incessant ventricular arrhythmias, diabetes mellitus with end organ damage, ongoing pregnancy, or contraindication to contrast-enhanced CMR including pacemakers, defibrillators, stage IV/V chronic kidney disease. All patients had an established diagnosis of HCM defined by an unexplained LV wall thickness of > 15 mm [19]. The dataset included demographic data, CMR imaging, biomarkers and genetic data with Doppler echo assessment [20].

CMR image acquisition was performed on General Electric, Philips and Siemens scanners, at either 1.5 or 3.0 Tesla, using multi-channel, phased-array chest coils and electrocardiographic gating. Short-axis cine steady-state free precession (SSFP) imaging was performed in slices of 8 mm thickness to cover the entire heart followed by long axis SSFP cine imaging. Cine SSFP parameters comprised TR/TE 3.1/1.2 ms, in-plane resolution of 2–2.5 mm and temporal resolution of 40–50 ms. Parasternal long axis 3CH view CMR cine images were considered in this study.

Echocardiographic data was obtained from routine clinical care closest to the CMR acquisitions (median time between scans was 2 months, interquartile range was 6 months, with 35% of cases acquired the same day), and included continuous-wave Doppler acquisitions to determine the LVOT pressure drop, which was estimated through the simplified Bernoulli formulation [21]. A resting pressure drop of ≥ 30 mmHg was taken as indicative of LVOTO (oHCM group); patients with < 30 mmHg were allocated into the non-obstructive group (nHCM).

### Manual landmark annotation

A standard operating procedure (SOP) was developed to standardise the manual labelling of anatomical landmarks in CMR images using ITK-Snap version 3.8.0 [22]. Five frames were annotated such that all frames showing maximum obstruction were tracked. The end-systolic frame was identified by the frame in which the LV was maximally contracted and where the mitral valve (MV) was fully closed. The mid-systolic frame was selected as the frame halfway between the first frame and the end-systolic frame and two further frames were annotated two frames before and after the mid-systolic frame. Lastly, the end-diastolic frame was identified as the frame in which the LV appeared to be maximally dilated and where the MV was open.

**Fig. 2:**
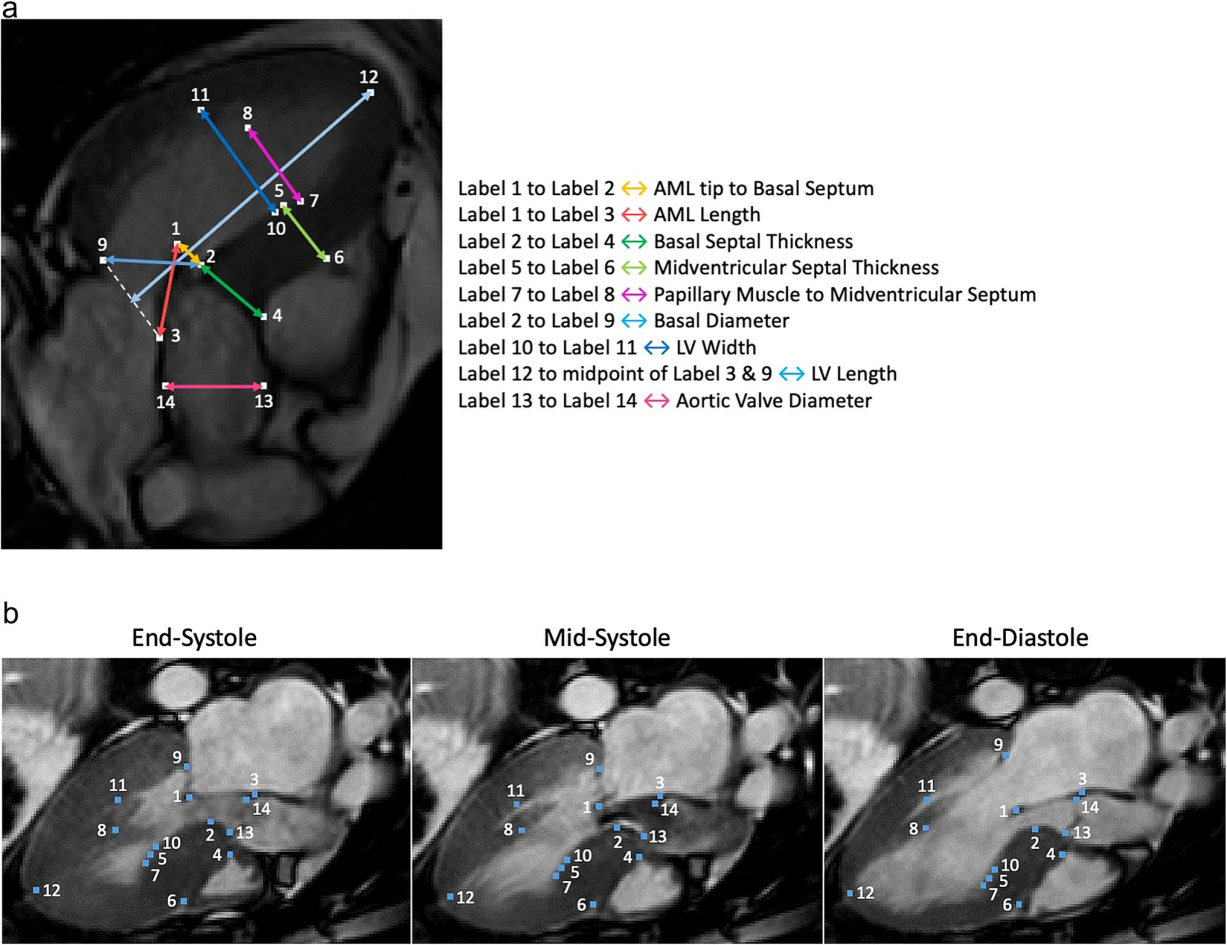
Anatomical metrics based on the collection of 14 landmarks. Panel **A**: Anatomical metrics defined as distances between landmarks (end-diastole). Panel **B**: The 14 landmarks in 3 key frames (different case from panel A, illustrating how image orientation is different between cases)

In total, 14 landmarks were identified, as summarised in Fig. 2, which allowed the following 11 anatomical metrics to be analysed: AML tip to basal septum (i.e. outflow tract dimension), AML length (the distance between the AML hinge and the AML tip), basal septal thickness, midventricular septal thickness, papillary muscle to midventricular septum, basal diameter, LV width, LV length (defined as the distance between the midpoint of the anterior and posterior mitral valve hinges and the apex of the heart on the epicardium), aortic valve diameter, AML length to LV width ratio and AML length to aortic valve diameter ratio.

Selected anatomical metrics were grouped according to three mechanisms identified in the literature: (i) septal hypertrophy, (ii) AML anatomy, and (iii) LV cavity morphology. Septal hypertrophy was captured by thickness at the base and at mid-ventricle. AML anatomy included AML length, length to LV width ratio, and AML length to aortic valve diameter ratio. Since the distance from AML tip to the basal septum quantifies the obstruction gap directly, this was not included in the individual mechanism groupings. LV cavity morphology was captured by LV length, and LV width at mid-ventricle (approximately half way between the apex and base, avoiding the papillary muscle). Also, the distance between the anterior papillary muscle and the septum was included in parallel with the LV width. Finally, the basal diameter was captured by two landmarks at the PML hinge and the basal septum.

Overall, 192 randomly selected cases were annotated with all 14 landmarks by three observers (MS, SRS, AWM), taking 30–35 min per case on average.

### Network design and training

Details of the network design and training scheme are given in the Supplementary Material. Briefly, landmarks were detected in two stages. Firstly, a region-of-interest (ROI) network was trained to detect four landmarks (numbers 5,11,12 and 13 in Fig. 2) on the full field of view images, resampled to a pixel resolution of 1.25 mm per pixel. The resulting landmarks were used to reorient the image into a standard orientation, with the LV long axis in the vertical orientation, and the resulting images were cropped based on the apex-to-base length. A second network was used to detect all 14 landmarks in the reoriented view, and the results transformed back to the original image dimensions. Landmarks were converted to heatmaps by convolving with a Gaussian of width 10 pixels for the ROI network and 6 pixels for the full landmark network. A UNet architecture was used to predict the heatmaps, and after prediction the maximum for each predicted heatmap was used as the landmark location.

### Quality control

A quality control post-processing step was implemented to highlight possible outliers amongst the model predictions and facilitate manual visual assessment of results. Firstly, temporal consistency of the predicted landmarks was assessed by computing the variance of all 11 derived distances for each anatomical metric across all frames in each case. The square root of the sum of the 11 variances for each case was found to obtain a single temporal quality score. Secondly, spatial consistency of the predicted landmarks was assessed by computing the distance between each of the 14 landmarks and the centre of mass in the five frames of interest. These 70 distances were normalised by the LV length in the end-diastolic frame to account for varying heart size. The mean distance between each landmark and the centre of mass was computed across all cases in each of the five frames. The variance between each distance and the mean value for each landmark was then found and all 70 variances were summed to obtain a single spatial quality score for each case.

Using the temporal and spatial quality assurance scores, the cases were ranked according from high to low scores. A manual visual assessment was then performed, through which outlier cases were discarded if one or more landmarks were misplaced in one or more frames.

### Statistics

Continuous variables were expressed as the mean ± standard deviation for each anatomical metric. Normality of the variables was tested for using the Chi Square test for normality to determine the goodness of fit of the data against a normal distribution at a 95% confidence level. For continuous and normally distributed data, an unpaired, two-tail Student’s t-test was used to compare the metrics between the nHCM and oHCM cohorts. For non-parametric data, a two-tail Mann–Whitney *U* test was used. p values of < 0.05 were considered statistically significant.

The inter-observer reproducibility was assessed in 20 randomly selected cases by determining the difference between the anatomical metrics across the three observers. Following a 3-week blanking period, the same 20 cases were annotated again by one observer to assess the intra-observer variability. The network predictions were considered as an additional observer to compare with the human observers. Inter- and intra-observer agreement for the measurements were evaluated using intraclass correlation coefficients (± 95% confidence intervals [CIs]). Typically, ICC < 0.4 is interpreted as poor agreement, 0.4–0.59 fair, 0.6–0.74 good, and 0.75–1.0 excellent [23]. Analysis was performed in MATLAB using the Statistics and Machine Learning Toolbox [24] (Mathworks, Natick, MA, USA).

Univariate linear regression analysis was performed to quantify the association between each individual metric and the LVOT Doppler pressure drop. The R^2^ coefficient of determination served as a measure to explain the proportion of variance in the LVOT pressure drops that could be explained by the different metrics.

We then evaluated the ability of all anatomical distances to predict the obstruction. Stepwise multivariate linear regression was performed using the Statistical Package for Social Sciences (SPSS) for Windows (ver. 27.0; SPSS Inc., Chicago, IL, USA). The SPSS stepwise regression model started with zero predictors and the most influential predictors of LVOTO were added sequentially with a significance of p < 0.001 and removed if p > 0.100. Then a logistic regression model (Model 1) was developed using the variables identified by the stepwise regression, with a tenfold cross validation method to evaluate the goodness of fit.

Furthermore, to investigate the mechanisms of LVOTO, selected anatomical metrics were grouped together according to three possible mechanisms: septal wall thickness, AML anatomy and LV cavity morphology. Accordingly, four additional logistic regression models were evaluated: Model 2 with all anatomical metrics excluding the AML tip to basal septum distance, and then one for each individual group.

Receiver Operating Characteristic (ROC) curve analysis was performed and the Area Under the Curve (AUC, or c-statistic) was computed which served as a baseline to compare the relative strength of association with LVOTO. The fast DeLong’s algorithm was used to estimate the AUC confidence intervals at 95% [25] and optimal cut-off values for each metric (in mm) for classifying obstruction were found by the Youden’s index which maximised both the specificity and sensitivity values [26].

## Results

### Study population characteristics

The study population characteristics are summarised in Table 1. Of the 2667 cases with adequate 3CH CMR, 2629 cases were processed in the network pipeline. Upon visual assessment of high temporal and spatial quality assurance scores, 34 cases were identified as outliers and thus were discarded from the final cohort. Due to missing resting Doppler data, 1905 cases were accessible in this study, resulting in 1,427 non-obstructive (75%) and 478 obstructive (25%) cases. Example videos of obstructive and non-obstructive cases are given in the Supplementary Materials.

**Table 1:** Study population baseline characteristics

	HCMR			p value
	Total (n = 1905)	nHCM (n = 1427)	oHCM (n = 478)	
Age, yrs	50 ± 11	49 ± 11	52 ± 10	2.67E−05
Male	1348 (71%)	1056 (74%)	292 (61%)	7.78E−08
Weight, kg	88.2 ± 18.7	87.4 ± 17.9	90.4 ± 20.9	2.67E−02
Height, m	173.3 ± 9.82	173.7 ± 9.75	172.1 ± 9.95	1.50E−03
BMI, kg/m^2^	29.3 ± 5.60	28.9 ± 5.29	30.4 ± 6.31	1.60E−05
NYHA class				
I	1174 (63%)	950 (68%)	224 (48%)	3.25E−18
II	548 (29%)	381 (27%)	167 (36%)	
III/IV	149 (8%)	73 (5%)	76 (16%)	
Hypertension	709 (37%)	502 (35%)	207 (43%)	1.50E−03
Diabetes Type 1	8 (0.4%)	6 (0.4%)	2 (0.4%)	9.95E−01
Diabetes Type 2	148 (8%)	103 (7%)	45 (9%)	1.22E−01
Pressure Drop (mmHg)	23.1 ± 31.0	8.26 ± 7.26	67.3 ± 32.4	3.95E−236

Values are presented as mean ± SD or n (% with respect to number of valid cases to account for missing data)

*HCM* Hypertrophic Cardiomyopathy, *nHCM* Hypertrophic Non-Obstructive Cardiomyopathy, *oHCM* Hypertrophic Obstructive Cardiomyopathy, *BMI* Body Mass Index, *NYHA* New York Heart Association

### Variability evaluation

**Fig. 3:**
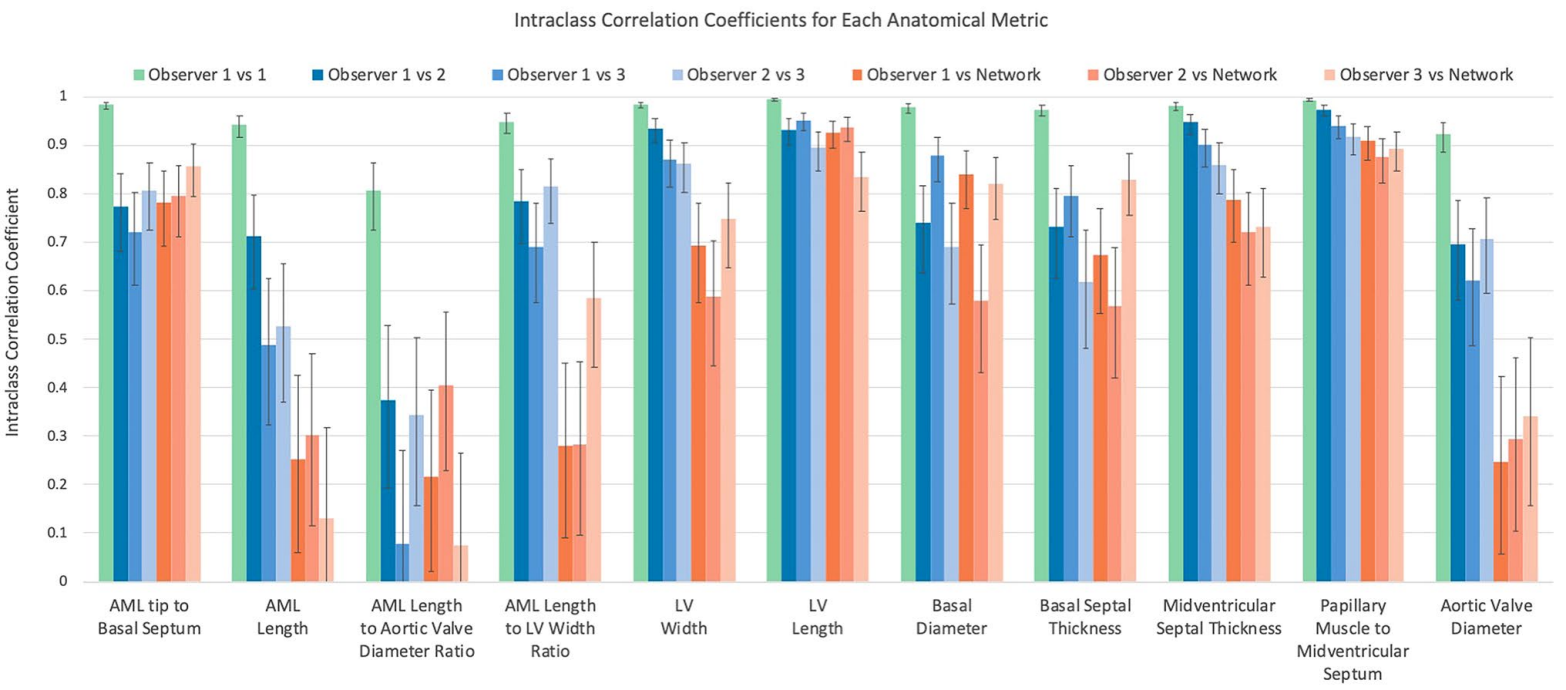
Bar chart showing the intraclass correlation coefficients for the intra- and inter- observers and network performance. Bars represent the intraclass correlation coefficient and error bars represent the 95% CI

Intraclass correlation coefficients (ICC) for intra- and inter-observer errors are shown in Fig. 3 and detailed in the Supplementary Materials Table S1. Of the 11 anatomical metrics, midventricular septal thickness, papillary muscle to midventricular septum, LV width and LV length had consistently excellent agreement (0.75–1.0 for all comparisons). Basal septal thickness, AML tip to basal septum, aortic valve diameter, basal diameter, and AML length to LV width ratio showed good to excellent ICC (over 0.6). AML length and AML length to aortic valve diameter ratio showed poor to fair agreement (< 0.6). The relatively poor agreement for AML length and AML length to aortic valve diameter ratio is likely due to the difficulty in visually assessing the AML tip position in the 3CH images.

The agreement between the network prediction and observers is shown in Fig. 3 and detailed in the Supplementary Materials Table S2. Confidence intervals overlapped with inter-observer confidence intervals in most cases. Similar to the inter-observer agreement, agreement between the observers and the network was good to excellent for midventricular septal thickness, papillary muscle to midventricular septum, LV length, and AML tip to basal septum (ICC > 0.6 with all three observers). Network agreement was fair to excellent for basal septal thickness, LV width, and basal diameter. However, AML length, aortic valve diameter AML length/LV width ratio, AML length/aortic valve diameter ratio were poorly predicted. This is likely due to the ambiguity in pixel neighbourhood patterns for precisely locating the AML tip and aortic valve landmarks, due to limited spatial and temporal resolution.

### Metric comparisons in the oHCM and nHCM cohorts

Average anatomical metrics are compared between oHCM and nHCM groups in the Supplementary Material Table S3. Taking a conservative Bonferroni correction for 55 multiple comparisons (p < 9.0E − 04), the oHCM group showed greater basal septal thickness, greater midventricular septal thickness during systole, smaller AML tip to basal septum distance, longer LV length, across all frames measured. AML length and AML length/aortic valve diameter ratio were larger at ED, and aortic valve diameter was smaller during systole, in the oHCM group.

### Univariate relationships

The univariate regression analysis for prediction of LVOT pressure drop as a continuous variable (Supplementary Material Table S4) showed significant relationships (p < 9.0E − 04) with basal septal thickness, midventricular septal thickness, AML tip to basal septum, Papillary muscle to midventricular septum, LV length, aortic valve diameter, basal diameter and AML length/LV width ratio at mid-systole and end-diastole. In addition, AML length and LV width was significant at mid-systole but not end-diastole.

The AUC for univariate predictors showed the highest AUC for AML tip to basal septum distance (0.80 at mid-systole, with a sensitivity of 74% and specificity of 74%). Next highest was basal septal thickness (mid-systole 0.67, 65% and 59% respectively) and LV length (mid-systole 0.64, 54% and 66% respectively).

### Multivariate relationships

Results of AUC for multivariate models are shown in Supplementary Table S6. In Model 1, all metrics in all frames were considered in the multivariate stepwise regression. The 8 significant anatomical metrics found were entered into the logistic regression model including: AML tip to basal septum in mid-systole, mid-systole − 2 and mid-systole + 2 frames; LV length in mid-systole + 2; basal diameter in mid-systole + 2; AML length to aortic valve diameter ratio in end-diastole; AML length to LV width ratio in mid-systole + 2; midventricular septal thickness in end-systole.

In model 2, all metrics in all frames were considered in multivariate stepwise regression, excluding AML tip to basal septum distance in all frames as it directly impacts on pressure drop. The 9 significant anatomical metrics found were entered into the logistic regression model including: basal septal thickness in end-diastole; AML length to aortic valve diameter ratio in end-diastole; AML length to LV width ratio in mid-systole + 2; LV width in mid-systole − 2; LV length in mid-systole + 2; basal diameter in mid-systole + 2; papillary muscle to IVS in end-diastole and mid-systole − 2; aortic valve diameter in mid-systole − 2.

**Fig. 4:**
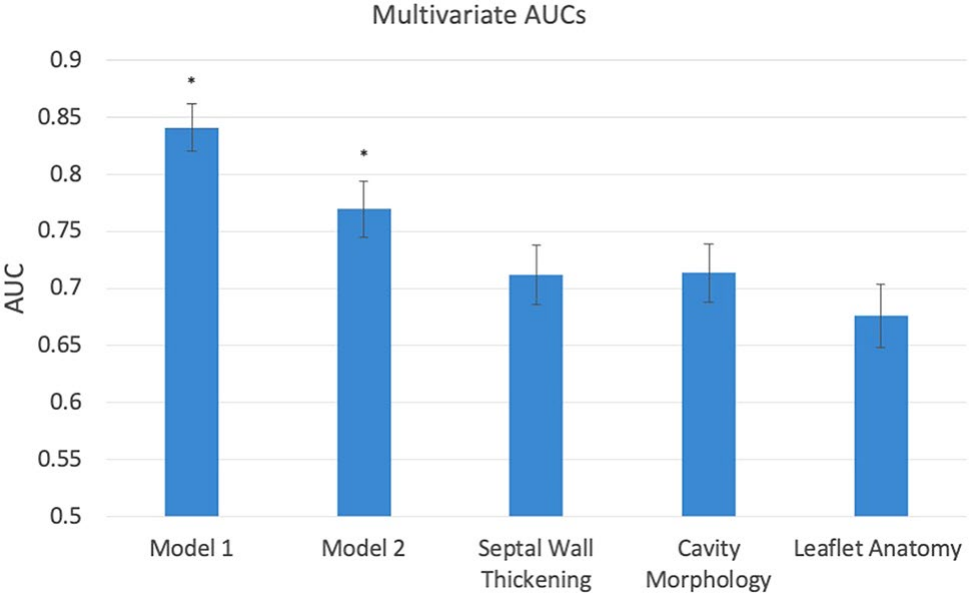
Ability of anatomical markers to predict the presence of LVOTO. Model 1 includes the anatomy of the obstruction, i.e. the AML to BS distance, whereas Model 2 excludes it. The other three bars report the ability of individual mechanisms to predict the presence of obstruction. Error bars show 95% confidence interval in the AUC. *p < 0.05 between model AUCs

In order to compare different possible mechanisms of LVOTO, the anatomical metrics were grouped into three main types: (a) septal hypertrophy (basal septal thickness, midventricular septal thickness), (b) AML anatomy (AML length, AML length to LV width ratio, AML to aortic valve diameter ratio); (c) cavity morphology (LV width, LV length, basal diameter). Anatomical metrics associated with each of these mechanisms were evaluated in separate logistical regression models and shown in Fig. 4.

Figure 4 shows that Model 1 performed significantly better than the others. Model 2 also performed significantly better than the three mechanistic groupings. However, the three mechanistic groupings performed similarly (no significant differences). Thus, a combination of all three mechanisms was significantly more associated with Doppler pressure drop than each mechanism alone.

## Discussion

A better understanding of the mechanisms underlying obstruction in HCM will enable better criteria to plan and monitor therapies that aim to decrease the obstruction. Here, we defined and automatically quantified anatomical metrics related to LVOTO from 3CH CMR images. The methods can be applied automatically to standard acquisitions and are available from www.cardiacatlas.org. The strongest relationships with Doppler pressure drop were exhibited by the mid-systolic AML to basal septum distance, as expected since this distance is highly related to the vena contracta of the outflow tract and hence the pressure drop estimated from the Bernoulli method. However, other metrics grouped according to the geometric mechanisms of septal hypertrophy, LV chamber geometry and AML anatomy were also highly associated, to a similar extent, with the presence of obstruction. In particular, basal septal thickness and LV length were strong predictors of obstruction. Also, a combination of mechanisms gave significantly stronger associations with Doppler pressure drop than each of the septal hypertrophy, LV cavity or AML leaflet anatomy mechanisms alone, indicating that each of these mechanisms contribute independent information.

### Human ability to label and measure distances

The distances measured between landmarks were consistent across different observers in 9 out of 11 metrics. All 4 metrics of the LV bulk anatomy (PM to IVS distance, IVS thickness, LV width, and LV length) reported ICC values > 0.90 that are considered to be of excellent reliability [27]. The metrics closer to the LVOT experienced a good reliability (ICC > 0.7 for AML to basal septum, aortic valve diameter, Basal Diameter and Basal Septal Thickness), and the metrics specific of the valve anatomy reported a low reliability (ICC < 0.7 for AML length and AML length to aortic valve diameter ratio) except for AML length to LV width ratio (ICC > 0.7).

The main reason for the low reliability of valve metrics is attributed to the relatively poor definition of the anatomy of the valve leaflet in the image. The resultant turbulent flow and movement of the AML can cause a distorted appearance on CMR. This is often exacerbated by an elongated AML, which is also a common morphological finding in HCM that contributes to SAM [28].

A more detailed analysis of the individual landmarks in CMR images revealed a reasonable consistency between the three independent observers with an average absolute error of 2.7 mm ± 0.6 mm. There were nevertheless differences across them: landmark 12, the apex of the heart, exhibited the least variation (1.6 mm), whereas landmark 10, at the lateral wall as one of the points to define LV width, exhibited the greatest variation (4.1 mm). These differences are caused by the room for subjectivity when identifying the landmarks defined in the SOP: whereas the apex is typically clearly visualised on 3-chamber long-axis cardiac CMR, and thus is an unambiguous landmark, the landmark 10 is not uniquely defined along the myocardium (i.e. different observers will place it at a variable height of the lateral wall). Similar difficulties are encountered with the basal septal thickness labels. Despite this ambiguity, the distances between pairs of landmarks reported good to excellent reliability: thicknesses and diameters are not heavily affected by their manual assessment at a variable location along the length of anatomical structures.

### Machine ability to learn from human annotations

The performance of the landmark detection network is instrumental to this study, as it allows to study morphological determinants of LVOTO in a large cohort in a systematic manner. The machine reproduced the human performance in 5 out of 9 metrics: the excellent reliability of 2 out of 4 LV bulk metrics (LV length and PM to IVS distance) and the good reliability of 3 out of 5 metrics close to the LVOT (AML tip to BS, basal diameter and basal septal thickness). One reason for the robustness of the LV length measurement is that it was calculated from three separate landmarks: the more data that is provided to the model, the better its performance will be [29].

By contrast, machine performance showed a sharp decline in two metrics (AML length to LV width ratio and aortic valve diameter) and a small decline in LV width and midventricular septal thickness. This is likely due to the difficulty in identifying the AML length in CMR 3CH images.

### Understanding obstruction

In a 3CH view the anatomy of the LVOT obstruction can be assessed by the AML to basal septum distance. In our study this is the distance that reported to be the most powerful independent predictor of LVOTO (AUC of 0.796 at mid-systole) and to negatively correlate between the with the degree of obstruction (R^2^ value of 0.189, p < 0.001).

One mechanistic factor that contributes to obstruction is the valve anatomy. A shortened AML to basal septum distance can be partially attributed to an elongated AML. This is a highly specific morphological change that occurs in HCM [30]. The elongated leaflet could amplify the effect of SAM by bringing the tip of the leaflet closer to the basal septum. This would create a narrower stenosis through which fast flowing blood leaves the left ventricle. The result is a drop in pressure, known as the Venturi effect [31], which causes the AML to be pulled anteriorly into the LVOT. Given the ability of SAM to predict obstruction in HCM as demonstrated by Nara et al. [18], this may explain why our results indicate AML anatomy metrics are highly predictive of LVOTO, despite relatively poor identification performance.

Mitral leaflet anatomy was examined by Maron et al. [28], finding that diastolic AML was elongated in HCM compared to controls but was not significantly associated with obstruction (27 ± 4 vs. 26 ± 5 mm, p = 0.57). Our results show similar mean values but the difference was significant in oHCM vs nHCM (27.1 ± 3.20 vs. 26.1 ± 3.20, p < 0.001, Table S3). The reason might be a combination of our larger statistical power (n = 1905 vs. n = 172 in [28]) and our improved accuracy in measurements through our deep learning solution (reduced standard deviation with a remarkable similarity in the mean values). On the other hand, Maron et al. [28] found that the ratio of AML to the width of the LV outflow tract was discriminant of the presence of LVOTO, in concordance with the current study (diastolic AML length to aortic valve diameter ratio, Table S3). It is important to note that absolute or relative AML length is still a very challenging metric from the 3CH views, especially when its relative size is considered (lower inter-observer ICC values in Fig. 3) and flow artefacts are present during systole. Further research to capture this metric in a reliable manner is thus warranted. Also, our study did not include the PML length, despite the evidence of its larger dimension in HCM compared to controls [26], due to the extra difficulty in observing this in the 3CH views.

A second mechanistic factor in the LVOTO is the basal septal thickness, that is reported to be a significant predictor and positively correlated with the obstruction degree (R^2^ value of 0.102 p < 0.001, at the frame before mid-systole). Clinically, and in previous studies, basal septal thickness has been known to be among the best predictors for LVOTO since obstruction tends to be more severe in patients with focal basal septal hypertrophy [17], as explained by the bulging of the septum into the LVOT, with a thickness of 16 mm generally used as a threshold [4].

The third mechanistic factor in the genesis of the obstruction is the bulk LV anatomy. LV length, which might be thought to have no link with obstruction, exhibited an acceptable AUC of 0.64, and the multi-variate model built with the 4 cavity morphological features achieved a similar performance to wall thickness (AUC of 0.714). A three-dimensional analysis of LV shape may offer additional insights into anatomical features influencing obstruction.

Although Doppler echo pressure drop is the preferred method for identification of LVOTO, many patients have poor characterization due to inadequate windows and limitations in the Bernoulli estimation. A 3CH CMR evaluation, able to identify patients with higher likelihood of LVOTO from anatomical metrics, may become a valid alternative providing valuable insights about the mechanisms causing the obstruction.

### Effect of temporal transients

In general, among metrics with significant differences during systole, the mid-systolic frame was representative of the differences so could be used if a single frame metric is required. We computed two additional logistic regression models, Model 3 and Model 4, which were similar to Model 1 and Model 2 respectively, except that metrics at frames ± 2 from mid-systole were ignored. Model 1 and Model 3 were not significantly different, and also Model 2 and Model 4 were not significantly different.

## Limitations

Other mechanisms of LVOTO include regional wall motion abnormalities, papillary muscle function, presence of apical HCM, malcoaptation of the mitral valve leaflets leading to mitral regurgitation, or conduction abnormalities. The landmarks captured were limited to the 3CH view, which despite being ideal in terms of providing a simple single view prediction of oHCM, meant that certain information could not be captured, including the aortoseptal angle which has been shown to correlate with LVOTO [32]. Moreover, studies using three-dimensional transoesophageal echocardiography and multidetector computed tomography have suggested that the LVOT is elliptical in shape, not round [33]. Therefore, the 2D LAX view may result in the underestimation of the LVOT diameter as it does not always portray the longest span of the outflow tract, thus this may have introduced a systematic error in these measurements [18].

While the 3-chamber LAX view was ideal for visualising the AML and septal hypertrophy, it often provided poor visualisation of the anterior papillary muscle. Incorporating the short axis view of the heart into the study would provide better representation of the papillary muscle morphology, which is thought to play an important role in LVOTO [17].

Phase contrast flow images were not investigated in this study. Also, the presence of flow artefacts in the CMR images often made the identification of the AML tip challenging. Flow artefacts commonly occur during systole due to fast and turbulent blood flow in the LVOT. This results in an increased signal phase shift, leading to intra-voxel phase dispersion and a loss of signal; hence the black appearance of the flow artefacts [34]. Thus, the visualisation of the AML tip was sometimes obscured hence the label positions were estimated by tracking the position of the AML from adjacent frames in which it was visible, which may have introduced subjectivity in its positioning. In addition, AML length is measured as a distance between two labels, however, as it is not straight in all of the frames, this can lead to the underestimation of its length. This is apparent during systole, especially in patients with SAM of the mitral valve, which causes the leaflet coaptation to move into the LVOT due to the drag phenomenon and the Venturi effect [16].

A further practical limitation of the study was that the findings were compared with resting LVOT pressure drops, obtained from Doppler TTE which were generally not measured on the same day as when the CMR acquisitions were performed, and with potential medical interventions between measurements. Due to the transient nature of LVOTO, the time interval between when the CMR and echocardiographic acquisitions were completed, as well as the pressure drop during physiological stress, could have significant implications on the predictive value of the distance measurements. Future work should investigate relationships between resting geometry and stress induced obstruction, using a more comprehensive analysis of 3D shape, which can be obtained from multiple cine views. Since appropriate echo and CMR data were only available for 1095 out of 2667 cases, selection bias cannot be ruled out.

Finally, studies have identified SAM of the MV as a significant morphological determinant of LVOTO [18], which is conventionally detected using echocardiography [15] as it is not directly measurable using CMR. Thus, it was not feasible to incorporate direct assessment of SAM into our SOP. However, morphological features closely related to the development of SAM were included in this study—in particular, the basal septal thickness and AML length.

## Conclusions

3CH CMR cine slices, typically acquired in all CMR exams, can be used to obtain anatomical metrics which identify patients with higher risk of LVOTO, suggesting further investigation if necessary in patients with high scores. Furthermore, metrics grouped into septal hypertrophy, LV geometry, and AML anatomy mechanisms each show strong associations, with the combination providing significant improvement, indicating that LVOTO is related to a combination of geometric factors. Network code and executables, as well as protocols for annotations, are available from www.cardiacatlas.org

## Supplementary Information

Below is the link to the electronic supplementary material.

Supplementary file1 (MP4 88 KB)

Supplementary file2 (MP4 110 KB)

Supplementary file3 (DOCX 105 KB)

## Data Availability

The access to the analysis pipeline (source code and executables) and to the segmentation operation procedure (SOP) is openly provided at cardiacatlas.org.
