# Supplementary material for: Machine learning evaluation of LV outflow obstruction in hypertrophic cardiomyopathy using three-chamber cardiovascular magnetic resonance
Source: Int J Cardiovasc Imaging. 2022 Oct 6;38(12):2695–705. doi: 10.1007/s10554-022-02724-7 (PMC9708771; doi:10.1007/s10554-022-02724-7)
Supplement: Supplementary file 3 — Supplementary file3 (DOCX 105 KB) [file 10554_2022_2724_MOESM3_ESM.docx]

# Supplementary Material

## Data Preparation

The parasternal LAX, 3 chamber view CMR cine images were resampled to a fixed 1.25 mm pixel spacing and cropped or padded to 256x256 pixels before input into the network due to varying acquired fields-of-view across patients. The image intensity was normalised to the range of [0,1] to retain the image intensities within 1% to 99% of the intensity range. This allowed for a more uniform intensity distribution by removing pixels with extreme dark or bright intensities.

## Networks

The Region of Interest (ROI) network was implemented to standardise the heart orientation in the image, using four landmark positions (landmarks 5, 11, 12 and 13 in Figure 2, including the AML hinge, apex, PML hinge and the inferior aortic valve landmark) to align the LV axis to a vertical position along the centre of the image. During training, image augmentations were applied, such as rotation by ± 180^o^, flipping and translation to allow the network to learn a large variation of anatomy. Intensities were augmented to between the 80th and 100th percentiles of the original image intensity to allow the network to learn a range of intensities. The landmarks for the apex and AML hinge were used to create a bounding box to crop the images to retain only the ROI and the cropped images were resized to 128 x 128. The mid-point of distance between the apex and AML hinge landmarks was used as the centre of the bounding box and 150% of the length between the landmarks was used as the size of the bounding box to ensure that all the relevant anatomy was included in the final image.

The cropped images were used as the input to a second landmark detection network which predicted all 14 landmark locations. Image intensities were augmented to between the 90th and 100th percentiles of the original image intensity. After landmark detection, the landmark locations were transformed back to the size and orientation of the original input image.

## Implementation

The ROI and landmark detection tasks were formulated as a heatmap. Each landmark in the manually annotated images was convolved with a Gaussian filter kernel of standard deviation 𝜎 = 10 pixels for the ROI network and 𝜎 = 6 pixels for the landmark detection network. The subsequent blurred distribution represented the spatial probability of the landmark occurring in that position. The landmark detection task was thus converted into a semantic segmentation problem, where the background was represented by one class and each landmark was represented by another class. We used a variation of the 2D CNN U-net architecture (35).

Five-fold cross validation was used to train the networks and for the ROI network, the dataset was randomly divided into a training set of 90 cases, containing the 4 landmarks used for image reorientation and the test set consisted of 10 cases in each of the five folds. For the landmark detection network, the training and validation set consisted of 173 and 19 cases respectively, all of which had 14 landmarks. In all sets, the proportion of cases of oHCM and HNCM were equal and there was no overlap of cases between the training, validation, and test sets.

Batch normalisation was implemented during the training process (36) and the activation function used was the PReLU function with 𝛼 initialised to 0.25 (37). The loss function that was optimised during the training process was the mean square error (MSE). The networks were trained for 500 epochs using a batch size of 10 and the Adam optimiser was used to update the model parameters with a learning rate of 0.001 and default parameters 𝛽_1_= 0.9, 𝛽_2_ = 0.999 and epsilon = 1e^-8^. The CNN models were trained using the open-source foundation Medical Open Network for Artificial Intelligence (MONAI version 0.4.0) PyTorch-based framework and training was performed on a PC with an NVIDIA GPU graphics card (38). The training process lasted approximately 7 hours for the ROI network and 5 hours for the landmark detection network. The best model was selected as the model which minimised the MSE loss and resulted in the best performance on the validation set.

Image intensities were rescaled to the average of the intensity augmentation range which was the 90^th^ percentile for the ROI network and the 95^th^ percentile for the landmark detection network, so that the data was normalised to the centre of the training data intensity distribution. Bicubic interpolation was used to obtain predicted heatmaps and these were obtained for the entire cohort by applying the best trained model to the remaining dataset, taking around 9 seconds per case and the heatmaps were normalised to the intensity range [0 255]. An intensity threshold of 128 was applied to the normalised heatmaps and the centroid of the largest connected component was extracted to obtain the coordinates of the predicted landmarks. The five frames of interest were identified by calculating the LV width in each frame in the CMR sequence. The frame with the largest LV width corresponded to end-diastole, while the frame with the shortest LV width corresponded to end-systole. Hence, mid-systole was identified as the midway frame between the first frame and end-systole, and the final two frames were calculated as two frames prior to and after the mid-systolic frame. The Euclidean distances between landmark were calculated to obtain the predicted distance metrics.

The 2D U-net was used as the network architecture for the deep learning model since it is well-suited for biomedical image segmentation problems (35). Images were fed as an input tensor with a shape of (number of images) x (image height) x (image width) x (image channels) into the U-net architecture and were propagated through several hidden layers in the network, leading to the output segmentation.

The U-net architecture is composed of the encoder or contractive path and the decoder or expansive pathway. The encoder path acquires semantic or contextual information in the image and is composed of a standard CNN structure, with recurrent blocks of two 3x3 unpadded convolutional operations performed by kernels with batch normalisation (36). The convolutional layer resulted in a tensor of the shape (number of images) x (feature map height) x (feature map width) x (feature map channels). The PReLU was selected as the activation function to overcome the dying Rectified Linear Unit (ReLU) problem associated with the standard ReLU activation function, where all negative inputs are always mapped to zero, thus decreasing the ability of the model to learn (37). After convolutions, the PReLU activation function and 2 x 2 max pooling stride two operation are used to reduce the spatial resolution or height and width of the image in a process known as downsampling, which resulted in doubling of the feature channels. Downsampling is implemented to perform dimensionality reduction to decrease the complexity of the image by reducing the number of parameters in the network. This works by selecting the pixel value in every 2 x 2 block of the input feature map with the maximum value, thus a pooled feature map was obtained which preserves the most significant values in the image. The decoder or expansive pathway on the right-side regains spatial information thus enabling accurate landmark localisation. The decoder path involved upsampling the feature map to increase the feature dimensions, which ensures the feature map is the same size as the original input. Upsampling is followed by 2 x 2 up-convolution blocks, which doubles the size of the image and halves the number of feature channels. Concatenation is then used to crop the feature map due to loss of border pixels as a result of convolution. This is followed by two 3 x 3 convolutions and the activation function unit, which ensure low resolution features are learnt and projected onto a higher resolution pixel space to attain a dense classification. High-level resolution feature maps obtained from the encoder path are concatenated with upsampled features, thus the network learns better representations following convolutions. The layers are fully connected, and the final convolution layer output a tensor of shape (number of images) x (number of segmentation classes) containing a per pixel score of a pixel belonging to a class. This score was converted to a probability using the SoftMax function, where small or negative inputs were mapped close to zero and large or positive inputs were mapped close to one.


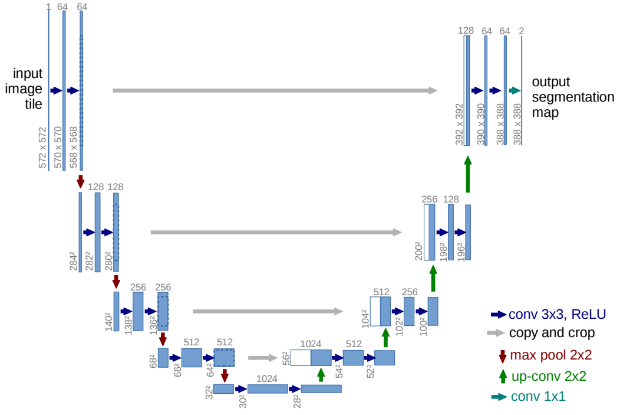


Fig. S1. U-net architecture where blue boxes represent multi-channel feature maps and white boxes represent copied feature maps. The number on top of the boxes represents the number of channels and the lower left corner of the boxes corresponds to the x-y size. The arrows represent different operations.

Table S1: **Intraclass correlation coefficients for intra- and inter- observer variability.** *Values presented as the ICC and the 95% CI.*

|  | Intra-observer | Inter-observer | | | |
| --- | --- | --- | --- | --- | --- |
|  | Observer 1 vs Observer 1 | Observer 1 vs Observer 2 | Observer 1 vs Observer 3 | Observer 2 vs Observer 3 | |
| **Basal septal thickness** | 0.974  (0.962-0.983) | 0.732  (0.626-0.811) | 0.796  (0.712-0.858) | | 0.617  (0.480-0.725) |
| **Midventricular septal thickness** | 0.982  (0.973-0.988) | 0.948  (0.923-0.964) | 0.900  (0.856-0.932) | | 0.860  (0.799-0.904) |
| **AML length** | 0.942  (0.916-0.961) | 0.713  (0.602-0.797) | 0.488  (0.324-0.624) | | 0.528  (0.371-0.655) |
| **AML tip to basal septum** | 0.984  (0.976-0.989) | 0.774  (0.682-0.842) | 0.721  (0.612-0.803) | | 0.806  (0.724-0.865) |
| **Papillary muscle to midventricular septum** | 0.993  (0.990-0.996) | 0.974  (0.961-0.982) | 0.941  (0.914-0.960) | | 0.918  (0.881-0.944) |
| **LV width** | 0.984  (0.977-0.989) | 0.934  (0.904-0.955) | 0.871  (0.815-0.912) | | 0.863     (0.803-0.906) |
| **LV length** | 0.995  (0.992-0.996) | 0.932  (0.900-0.954) | 0.952  (0.930-0.967) | | 0.895  (0.848-0.928) |
| **Aortic valve diameter** | 0.922  (0.887-0.947) | 0.697  (0.581-0.786) | 0.622  (0.486-0.729) | | 0.707  (0.594-0.793) |
| **Basal diameter** | 0.978  (0.967-0.985) | 0.740  (0.637-0.817) | 0.879  (0.826-0.917) | | 0.690     (0.572-0.780) |
| **AML length/LV width ratio** | 0.949  (0.925-0.965) | 0.786  (0.698-0.851) | 0.691  (0.574-0.781) | | 0.816  (0.738-0.8720 |
| **AML length/aortic valve diameter ratio** | 0.806  (0.725-0.865) | 0.373  (0.192-0.529) | 0.077  (-0.119-0.269) | | 0.343  (0.158-0.504) |

Table S2: **Intraclass correlation coefficients for the observers vs network.** *Values presented as the intraclass coefficient and the 95% CI.*

|  | Observer 1 vs Network | Observer 2 vs Network | Observer 3 vs Network |
| --- | --- | --- | --- |
| **Basal septal thickness** | 0.675  (0.553-0.769) | 0.569  (0.421-0.688) | 0.829  (0.756-0.882) |
| **Midventricular septal thickness** | 0.787  (0.699-0.851) | 0.721  (0.611-0.803) | 0.732  (0.627-0.811) |
| **AML length** | 0.253  (0.061-0.427) | 0.302  (0.114-0.470) | 0.130  (-0.066-0.317) |
| **AML tip to basal septum** | 0.781  (0.691-0.847) | 0.795  (0.711-0.857) | 0.857  (0.795-0.902) |
| **Papillary muscle to midventricular septum** | 0.909  (0.868-0.938) | 0.877  (0.823-0.915) | 0.894  (0.847-0.928) |
| **LV width** | 0.692  (0.574-0.781) | 0.589  (0.445-0.703) | 0.749     (0.648-0.823) |
| **LV length** | 0.927  (0.894-0.951) | 0.937  (0.907-0.957) | 0.835  (0.765-0.886) |
| **Aortic valve diameter** | 0.248  (0.056-0.423) | 0.294  (0.105-0.463) | 0.342  (0.157-0.503) |
| **Basal diameter** | 0.839  (0.770-0.888) | 0.579  (0.432-0.695) | 0.822     (0.746-0.876) |
| **AML length/LV width ratio** | 0.279  (0.089-0.450) | 0.284  (0.095-0.454) | 0.586  (0.441-0.701) |
| **AML length/aortic valve diameter ratio** | 0.216  (0.022-0.395) | 0.406  (0.229-0.557) | 0.074  (-0.123-0.265) |

Table S3: **Average measurement of each metric in each group.** *Continuous variables are presented as the mean ± SD.*

|  | Frame | oHCM group | nHCM group | *P* value |
| --- | --- | --- | --- | --- |
| **Basal septal thickness** | Mid Systole -2 | 19.0 ± 3.13 | 16.6 ± 3.20 | 2.65E-38 |
|  | Mid Systole | 19.5 ± 3.20 | 17.5 ± 3.08 | 3.92E-29 |
|  | Mid Systole +2 | 19.6 ± 2.85 | 17.9 ± 2.86 | 8.14E-25 |
|  | End Systole | 19.4 ± 2.74 | 18.0 ± 2.78 | 5.45E-21 |
|  | End Diastole | 18.0 ± 2.91 | 15.8 ± 3.09 | 4.87E-41 |
| **Midventricular septal thickness** | Mid Systole -2 | 18.8 ± 5.19 | 17.8 ± 494 | 1.08E-04 |
|  | Mid Systole | 20.0 ± 4.85 | 19.0 ± 4.71 | 2.91E-05 |
|  | Mid Systole +2 | 20.7 ± 4.53 | 19.9 ± 4.43 | 4.49E-04 |
|  | End Systole | 22.1 ± 4.61 | 21.0 ± 4.71 | 3.11E-07 |
|  | End Diastole | 16.4 ± 5.42 | 16.0 ± 5.82 | 9.29E-02 |
| **AML length** | Mid Systole -2 | 26.6 ± 3.15 | 26.3 ± 3.08 | 2.86E-01 |
|  | Mid Systole | 26.4 ± 3.92 | 26.6 ± 3.30 | 2.76E-02 |
|  | Mid Systole +2 | 26.5 ± 3.95 | 26.7 ± 3.26 | 4.10E-03 |
|  | End Systole | 27.1 ± 3.67 | 27.1 ± 3.31 | 7.53E-01 |
|  | End Diastole | 27.1 ± 3.20 | 26.1 ± 3.20 | 4.79E-10 |
| **AML tip to basal septum** | Mid Systole -2 | 15.1 ± 4.79 | 20.2 ± 5.05 | 1.06E-76 |
|  | Mid Systole | 13.5 ± 4.13 | 18.5 ± 4.87 | 7.18E-84 |
|  | Mid Systole +2 | 13.3 ± 3.96 | 17.7 ± 4.52 | 2.01E-78 |
|  | End Systole | 14.4 ± 3.68 | 17.3 ± 4.12 | 2.27E-39 |
|  | End Diastole | 13.4 ± 4.42 | 15.5 ± 5.23 | 1.62E-14 |
| **Papillary muscle to midventricular septum** | Mid Systole -2 | 22.6 ± 7.02 | 22.9 ± 7.61 | 4.62E-01 |
|  | Mid Systole | 18.9 ± 5.85 | 18.7 ± 6.67 | 2.38E-01 |
|  | Mid Systole +2 | 16.4 ± 4.92 | 15.7 ± 5.48 | 5.12E-04 |
|  | End Systole | 13.9 ± 3.85 | 13.9 ± 4.64 | 8.57E-02 |
|  | End Diastole | 29.9 ± 8.38 | 28.2 ± 8.06 | 7.84E-06 |
| **LV width** | Mid Systole -2 | 33.7 ± 6.69 | 34.5 ± 7.72 | 5.12E-02 |
|  | Mid Systole | 30.2 ± 5.89 | 30.1 ± 6.75 | 5.81E-01 |
|  | Mid Systole +2 | 27.6 ± 5.36 | 26.5 ± 5.83 | 2.99E-05 |
|  | End Systole | 23.2 ± 4.33 | 22.5 ± 5.21 | 8.52E-04 |
|  | End Diastole | 42.4 ± 7.54 | 41.1 ± 7.87 | 9.66E-05 |
| **LV length** | Mid Systole -2 | 101 ± 9.87 | 96.6 ± 9.55 | 5.74E-14 |
|  | Mid Systole | 98.9 ± 9.74 | 94.1 ± 9.27 | 3.17E-21 |
|  | Mid Systole +2 | 97.2 ± 9.77 | 92.1 ± 9.34 | 8.26E-24 |
|  | End Systole | 95.7 ± 10.1 | 90.8 ± 9.44 | 1.79E-21 |
|  | End Diastole | 103 ± 9.56 | 98.7 ± 9.49 | 1.71E-16 |
| **Aortic valve diameter** | Mid Systole -2 | 22.1 ± 2.84 | 22.6 ± 2.92 | 2.96E-04 |
|  | Mid Systole | 22.0 ± 2.87 | 22.6 ± 2.87 | 4.39E-05 |
|  | Mid Systole +2 | 22.1 ± 2.80 | 22.6 ± 2.87 | 2.34E-04 |
|  | End Systole | 22.0 ± 2.81 | 22.4 ± 2.80 | 7.60E-03 |
|  | End Diastole | 21.6 ± 2.75 | 21.9 ± 2.65 | 6.30E-03 |
| **Basal diameter** | Mid Systole -2 | 36.8 ± 6.62 | 38.3 ± 5.39 | 7.26E-07 |
|  | Mid Systole | 35.2 ± 5.09 | 36.4 ± 5.21 | 8.75E-06 |
|  | Mid Systole +2 | 33.8 ± 4.95 | 34.8 ± 5.05 | 5.57E-05 |
|  | End Systole | 33.5 ± 5.26 | 34.2 ± 5.14 | 1.30E-03 |
|  | End Diastole | 39.2 ± 5.31 | 40.3 ± 5.72 | 2.02E-04 |
| **AML length/LV width ratio** | Mid Systole -2 | 0.820 ± 0.192 | 0.806 ± 0.215 | 3.02E-02 |
|  | Mid Systole | 0.908 ± 0.218 | 0.928 ± 0.241 | 7.16E-02 |
|  | Mid Systole +2 | 0.994 ± 0.242 | 1.05 ± 0.246 | 1.24E-07 |
|  | End Systole | 1.21 ± 0.285 | 1.26 ± 0.320 | 9.78E-04 |
|  | End Diastole | 0.660 ± 0.155 | 0.659 ± 0.160 | 9.03E-01 |
| **AML length/Aortic valve diameter ratio** | Mid Systole -2 | 1.22 ± 0.184 | 1.17 ± 0.168 | 1.12E-04 |
|  | Mid Systole | 1.22 ± 0.231 | 1.19 ± 0.180 | 9.08E-02 |
|  | Mid Systole +2 | 1.22 ± 0.210 | 1.20 ± 0.175 | 3.28E-01 |
|  | End Systole | 1.25 ± 0.303 | 1.22 ± 0.178 | 2.99E-02 |
|  | End Diastole | 1.26 ± 0.154 | 1.20 ± 0.163 | 2.34E-15 |

Table S4: **Univariate linear regression analysis for prediction of the LVOT pressure drop.**

|  | **Frame** | **R^2^coefficient** | | ***P* value** |
| --- | --- | --- | --- | --- |
| **Basal septal thickness** | Mid Systole -2  Mid Systole  Mid Systole +2  End Systole  End Diastole | 0.102  0.081  0.068  0.054  0.104 | ^3.80E-15^  ^1.71E-11^  ^4.17E-10^  ^2.58E-7^  ^2.47E-15^ | |
| **Midventricular septal thickness** | Mid Systole -2  Mid Systole  Mid Systole +2  End Systole  End Diastole | 0.00839  0.00805  0.00575  0.0109  0.000215 | 9.89E-7  5.64E-5  0.000132  0.00938  2.83E-24 | |
| **AML length** | Mid Systole -2  Mid Systole  Mid Systole +2  End Systole  End Diastole | 0.00116  0.000338  0.00188  0.000434  0.0177 | ^0.0208^  ^6.30E-7^  ^1.80E-9^  ^0.00164^  ^0.0680^ | |
| **AML tip to basal septum** | Mid Systole -2  Mid Systole  Mid Systole +2  End Systole  End Diastole | 0.181  0.189  0.175  0.0920  0.0348 | ^2.51E-159^  ^3.58E-167^  ^9.41E-156^  ^6.36E-95^  ^9.45E-71^ | |
| **Papillary muscle to midventricular septum** | Mid Systole -2  Mid Systole  Mid Systole +2  End Systole  End Diastole | 0.000229  0.000245  0.00434  4.23E-5  0.0119 | ^3.92E-26^  ^7.93E-23^  ^2.36E-14^  ^1.38E-21^  ^1.25E-5^ | |
| **LV width** | Mid Systole -2  Mid Systole  Mid Systole +2  End Systole  End Diastole | 0.00133  0.000320  0.00900  0.00513  0.00828 | 4.14E-17  1.02E-9  3.34E-5  7.55E-5  0.0333 | |
| **LV length** | Mid Systole -2  Mid Systole  Mid Systole +2  End Systole  End Diastole | 0.0380  0.0442  0.0581  0.0527  0.0410 | ^1.19E-7^  ^2.88E-8^  ^3.89E-13^  ^3.18E-11^  ^9.19E-9^ | |
| **Aortic valve diameter** | Mid Systole -2  Mid Systole  Mid Systole +2  End Systole  End Diastole | 0.0105  0.00989  0.00819  0.00378  0.00275 | 1.08E-17  4.29E-17  1.36E-15  2.19E-11  5.34E-10 | |
| **Basal diameter** | Mid Systole –2  Mid Systole  Mid Systole +2  End Systole  End Diastole | 0.0190  0.0144  0.0112  0.00565  0.0105 | 5.36E-27  1.43E-22  3.29E-20   6.30E-16  5.24E-19 | |
| **AML length/LV width ratio** | Mid Systole -2  Mid Systole  Mid Systole +2  End Systole  End Diastole | 0.000304  0.00228  0.0162  0.00716  8.27E-5 | 1.91E-13  2.56E-23  3.02E-37  1.05E-29  1.96E-15 | |
| **AML length/aortic valve diameter ratio** | Mid Systole -2  Mid Systole  Mid Systole +2  End Systole  End Diastole | 0.0148  0.00530  0.00160  0.00529  0.0282 | 0.561  0.0389  0.00133  0.0119  0.00294 | |
|  |  |  |  |  |

Table S5: **Discriminatory performance of each anatomical parameter**. *Cutoff values (in mm), sensitivity and specificity computed as the maximal Youden index.*

|  | **Frame** | **AUC (95% CI)** | **Cutoff (mm)** | **Sensitivity (%)** | **Specificity (%)** |
| --- | --- | --- | --- | --- | --- |
| **Basal septal thickness** | Mid Systole -2  Mid Systole  Mid Systole +2  End Systole  End Diastole | 0.697 (0.671-0.724)  0.671 (0.644-0.698)  0.657 (0.629-0.685)  0.643 (0.615-0.672)  0.701 (0.675-0.728) | 16.9  18.3  18.9  18.7  18.0 | 76.6  64.9  59.6  60.9  52.5 | 50.7  59.1  63.1  59.8  76.4 |
| **Midventricular septal thickness** | Mid Systole -2  Mid Systole  Mid Systole +2  End Systole  End Diastole | 0.559 (0.530-0.588)  0.564 (0.535-0.593)  0.554 (0.524-0.583)  0.578 (0.549-0.607)  0.526 (0.496-0.555) | 16.6  17.7  19.0  20.9  14.6 | 63.6  68.6  64.0  58.8  56.9 | 46.0  42.7  46.9  54.9  48.4 |
| **AML length** | Mid Systole -2  Mid Systole  Mid Systole +2  End Systole  End Diastole | 0.516 (0.486-0.546)  0.466 (0.436-0.497)  0.456 (0.426-0.486)  0.505 (0.475-0.535)  0.595 (0.566-0.624) | 26.6  37.5  34.7  27.4  26.7 | 50.2  1.30  2.72  47.7  56.5 | 54.1  99.7  98.8  56.3  58.5 |
| **AML tip to basal septum** | Mid Systole -2  Mid Systole  Mid Systole +2  End Systole  End Diastole | 0.779 (0.756-0.803)  0.796 (0.773-0.819)  0.786 (0.763-0.809)  0.700 (0.674-0.727)  0.617 (0.589-0.645) | 16.9  15.3  15.1  15.5  15.7 | 73.7  73.7  70.6  65.2  45.5 | 69.0  74.3  76.4  64.4  75.1 |
| **Papillary muscle to midventricular septum** | Mid Systole -2  Mid Systole  Mid Systole +2  End Systole  End Diastole | 0.489 (0.460-0.518)  0.518 (0.489-0.547)  0.553 (0.525-0.581)  0.526 (0.498-0.555)  0.568 (0.538-0.598) | 16.6  12.4  12.0  10.9  30.4 | 79.9  89.5  84.5  80.5  53.1 | 23.8  17.5  27.3  29.6  60.8 |
| **LV width** | Mid Systole -2  Mid Systole  Mid Systole +2  End Systole  End Diastole | 0.468 (0.440-0.489)  0.508 (0.480-0.546)  0.564 (0.535-0.593)  0.551 (0.523-0.567)  0.559 (0.530-0.572) | 26.9  25.2  22.9  21.1  44.4 | 86.4  82.6  84.9  70.7  43.1 | 17.6  24.7  28.1  42.4  68.7 |
| **LV length** | Mid Systole -2  Mid Systole  Mid Systole +2  End Systole  End Diastole | 0.615 (0.585-0.644)  0.635 (0.606-0.664)  0.644 (0.615-0.673)  0.637 (0.608-0.666)  0.620 (0.591-0.649) | 99.8  97.7  94.5  94.4  103 | 54.0  54.4  60.3  54.0  49.2 | 63.6  65.9  60.7  66.3  67.6 |
| **Aortic valve diameter** | Mid Systole -2  Mid Systole  Mid Systole +2  End Systole  End Diastole | 0.553 (0.524-0.583)  0.556 (0.526-0.586)  0.550 (0.520-0.580)  0.536 (0.507-0.566)  0.542 (0.512-0.571) | 23.0  22.1  22.4  22.6  21.9 | 46.0  57.4  52.7  47.7  50.8 | 65.5  52.3  56.3  59.2  59.0 |
| **Basal diameter** | Mid Systole -2  Mid Systole  Mid Systole +2  End Systole  End Diastole | 0.593 (0.564-0.621)  0.568 (0.538-0.598)  0.562 (0.532-0.591)  0.549 (0.519-0.579)  0.559 (0.529-0.588) | 38.0  35.4  33.7  32.2  38.7 | 52.6  56.1  58.8  65.2  61.9 | 64.2  55.2  54.0  45.4  48.5 |
| **AML length/LV width ratio** | Mid Systole -2  Mid Systole  Mid Systole +2  End Systole  End Diastole | 0.533 (0.504-0.562)  0.473 (0.443-0.502)  0.419 (0.390-0.449)  0.450 (0.421-0.479)  0.502 (0.473-0.531) | 0.642  0.734  0.489  0.933  0.499 | 84.7  82.2  100  89.5  92.5 | 22.6  19.7  0.350  11.4  12.8 |
| **AML length/Aortic valve diameter ratio** | Mid Systole -2  Mid Systole  Mid Systole +2  End Systole  End Diastole | 0.559 (0.529-0.588)  0.526 (0.496-0.556)  0.515 (0.485-0.545)  0.533 (0.504-0.563)  0.621 (0.593-0.649) | 1.08  1.13  1.28  1.12  1.21 | 82.4  68.6  31.8  79.5  64.9 | 27.2  37.4  72.5  27.4  54.1 |

Multivariate Predictors:

Table S6. **Multivariate analysis for prediction of the LVOT pressure drop using logistic regression.** *Variables considered significant from multivariate stepwise regression analysis are entered in the multivariate models*

| **Logistic Regression** | **AUC (95% CI)** |
| --- | --- |
| Model 1: Including AML tip to BS | 0.841 (0.821 – 0.862) |
| Model 2: Excluding AML tip to BS | 0.770 (0.745 – 0.794) |
| Septal Wall Thickness | 0.712 (0.686 – 0.738) |
| Cavity Morphology | 0.714 (0.688 – 0.739) |
| Leaflet Anatomy | 0.676 (0.648 – 0.704) |

In model 3, all anatomical metrics were considered in multivariate stepwise regression, excluding those in the mid-systolic -2 and mid-systolic +2 frames in order to exclude temporal data. The 7 significant anatomical metrics found were entered into the logistic regression model including: AML tip to basal septum in mid-systole; LV length in mid-systole and end-diastole; LV width in end-diastole; basal diameter in end-diastole; AML length to aortic valve diameter ratio in end-diastole; basal septal thickness in end-diastole.

In model 4, all anatomical metrics were considered in multivariate stepwise regression, excluding those in the mid-systolic -2 and mid-systolic +2 frames in order to exclude temporal data, and also excluding AML tip to basal septum in all frames as it is directly associated with pressure drop. The 6 significant anatomical metrics found were entered into the logistic regression model including: basal septal thickness in end-diastole; AML length to aortic valve diameter in end-diastole; AML length to LV width in end-systole; LV length in end-systole; basal diameter in mid-systole; aortic valve diameter in mid-systole.

Table S7: **Multivariate analysis for prediction of the LVOT pressure drop using logistic regression.** *Variables considered significant from multivariate stepwise regression analysis are entered in the multivariate models.*

| **Logistic Regression** | **AUC (95% CI)** | **Accuracy (%)** | **Sensitivity (%)** | **Specificity (%)** |
| --- | --- | --- | --- | --- |
| Model 3: Including AML tip to BS, excluding ms-2, ms+2 | 0.834 (0.813 – 0.855) | 80.6 | 65.4 | 84.0 |
| Model 4: Excluding AML tip to BS, excluding ms-2, ms+2 | 0.764 (0.740 – 0.789) | 77.0 | 60.0 | 78.9 |

**HCMR Investigators**

NHLBI:

Patrice Desvigne-Nickens MD, Nancy Geller PhD, Dong-Yun Kim PhD

HCMR Site Investigators

| **Site** | **PI** |
| --- | --- |
| Cleveland Clinic | Milind Desai MD |
| Oxford University UK | Masliza Mahmod MD |
| Yale | Daniel Jacoby MD |
| Berlin – Charite, Germany | Jeanette Schulz-Menger MD |
| Calgary, Canada | James White MD |
| London - Kings College, UK | Amedeo Chiribiri MD, PhD |
| University of Michigan | Adam Helms MD |
| Northwestern University | Lubna Choudhury MD |
| Erasmus, Netherlands | Michel Michaels MD |
| Birmingham, UK | William Bradlow MD |
| University of Virginia Health System | Michael Salerno MD |
| Oregon University of the Health Sciences | Stephen Heitner MD |
| London – Royal Brompton, UK | Sanjay Prasad MD |
| London - Chest Hospital, UK | Saidi Mohiddin MD |
| Leeds, UK | Peter Swoboda MD |
| Tufts Medical Center | Martin Maron MD |
| Stuttgart, Germany | Heiko Mahrholdt MD |
| Bristol, UK | Chiara Bucciarelli-Ducci MD |
| Cornell | Jonathan Weinsaft MD |
| Duke | Han Kim MD |
| Leicester, UK | Gerry McCann MD |
| Amsterdam, Netherlands | Albert van Rossum MD |
| Mayo Clinic | Eric Williamson MD |
| Southhampton, UK | Andrew Flett MD |
| Aberdeen, UK | Dana Dawson MD |
| Montréal Heart Institute, Canada | F. Pierre Mongeon MD |
| Florence, Italy | Iacopo Olivotto MD |
| University of Toronto, Canada | Andrew Crean MD |
| University of Pennsylvania | Anjali Owens MD |
| Brigham | Carolyn Ho MD |
| London - St. Georges, UK | Lisa Anderson MD |
| Bologna, Italy | Elena Biagini MD |
| Edinburgh, UK | David Newby MD |
| Heidelberg, Germany | Matthias Friedrich MD |
| Glasgow, UK | Colin Berry MD |
| St. Luke’s Mt. Sinai | Bette Kim MD |
| Quebec City, Canada | Eric Larose MD |
| Johns Hopkins University | Theodore Abraham MD |
| New York University | Mark Sherrid MD |
| Beth Israel Deaconess | Evan Appelbaum MD |
| Methodist Hospital, Houston | Sherif Nagueh MD |
| Milan, Italy | Ornella Rimoldi MD |
| McGill University, Canada | Eleanor Elstein MD |
| Rome Sapienza, Italy | Camillo Autore MD |
